# Influence of Environmental Growth Factors on the Biomass and Pigment Production of *Chlorociboria aeruginascens*

**DOI:** 10.3390/jof5020046

**Published:** 2019-06-08

**Authors:** Stephanie Stange, Susanne Steudler, Hubertus Delenk, Anett Werner, Thomas Walther, André Wagenführ

**Affiliations:** 1 Faculty of Mechanical Science and Engineering, Institute of Natural Materials Technology, Technische Universität Dresden, Chair of Wood Technology and Fibre Materials Technology, 01069 Dresden, Germany; hubertus.delenk@tu-dresden.de (H.D.); andre.wagenfuehr@tu-dresden.de (A.W.); 2 Faculty of Mechanical Science and Engineering, Institute of Natural Materials Technology, Technische Universität Dresden, Chair of Bioprocess Engineering, 01069 Dresden, Germany; susanne.steudler@tu-dresden.de (S.S.); anett.werner@tu-dresden.de (A.W.); thomas_walther@tu-dresden.de (T.W.)

**Keywords:** *Chlorociboria aeruginascens*, xylindein, fungal pigments, fungal growth conditions, fungal polyketides

## Abstract

The soft rot fungus *Chlorociboria aeruginascens* produces a blue–green pigment xylindein, which is of considerable interest for various applications such as in the veneer industry or in organic semiconductors. To understand the fungal growth as well as pigment production of *C. aeruginascens*, several studies were performed, the results of which are presented here. These studies investigated various growth conditions such as temperature, pH value, oxygen level and light intensity. It was observed that the formation of xylindein by *C. aeruginascens* decoupled from growth. In the primary metabolismus, the uncolored biomass is formed. Pigment production took place within the secondary metabolism, while biomass growth as well as pigment production depended on various growth conditions. It was also found that certain conditions encourage the switch in metabolism, leading to pigment production.

## 1. Introduction

The fungus *Chlorociboria* sp. is well known as spalting fungi, which causes a blue–green discoloration in wood [1]. The pigment xylindein is responsible for the color appearance [1,2,3]. The focus of the study presented was not the spalting effect, but the production of the pigment xylindein as well as the fungal growth. Xylindein is produced by polykedite synthesis [4] and therewith belongs to the secondary metabolites of these fungi. Xylindein seems to have promising applicability as a fluorescent labeling agent or in organic semiconductor applications [5,6]. To date, it cannot be produced by chemical synthesis [7,8]. Hitherto, the only way to produce xylindein is through the extraction of the discolored biomass of *Chlorociboria* sp. or of fungal stained material such as blue–green wood. Therewith, a bioprocess engineered cultivation of the fungus would be a good opportunity to produce a sufficient amount of raw material. Harrison et al. [9] used 1 L of liquid batch bioreactors for the cultivation of *Chlorociboria aeruginosa* and the production of xylindein on a 2% malt medium (non-specification of the process parameters). This has been the largest scale experiment to date in the given current literature. To optimize the growth as well as the pigmentation and to get the cultivation to industry-relevant scales, the fungus, its metabolism, and its growth behavior have to be investigated in greater detail. Important influencing factors for the optimization are, on the one hand, the composition of the medium and, thus, the availability of the nutrients and, on the other hand, the process parameters, such as temperature, pH value or oxygen supply.

Therefore, in Stange et al. [10], the influence of various carbohydrate and nitrogen sources in the liquid culture medium on the growth as well as the pigmentation of *Chlorociboria aeruginascens* were described. There, the fungus used glucose, mannose, maltose, and xylose as carbohydrate source. As a nitrogen source, *Chlorociboria aeruginascens* preferred the tested organic nitrogen sources, especially yeast extract. Stange et al. [10] observed good biomass growth for high glucose and high yeast extract concentrations, but no pigmentation was documented for such media. [10] described, that under nitrogen limitation, xylindein production and therewith the blue–green discoloration of biomass or culture medium was visible. Therewith, the nitrogen content of the growth medium is one regulation factor for the syntheses of polyketides and, thus, for the pigmentation of biomass with xylindein [10]. Similar trigger effects for the production of fungal pigments via polyketide synthesis by a nitrogen limitation have been reviewed by Tudzynski [11]. In [11], a model of the effect of nitrogen limitation on the secondary metabolism of *Fusarium fujikuroi* for the production of the red pigment bikaverin is given. This model might be transferable for the secondary metabolism of *Chlorociboria* sp. and, thus, the production of xylindein.

Another preferred medium for the cultivation of *C. aeruginascens* regarding biomass as well as pigment production is orange juice, possibly because of its optimal ratio between carbohydrate and fungal available nitrogen [12]. Therefore, the tests were performed with an orange juice medium or rather orange juice agar.

Apart from a successful process strategy or the fungal available nutrients, the process parameters also have a certain influence on biomass and pigment production. In general, organisms react to stress. For instance, light, radiation, salty media or various pH values can act as stress factors on organisms. Many fungi react to these environmental conditions by producing secondary metabolites or spores [13]. Consequently, the aim of this study was to evaluate certain cultivation conditions such as light, pH value or oxygen supply, which have an influence on xylindein production of *Chlorociboria aeruginascens*.

## 2. Materials and Methods

For all experiments, the fungus *Chlorociboria aeruginascens* IHIA39 (of the International Institute (IHI) Zittau) was used. The fungus was maintained on orange juice agar plates (50% orange juice from Sonniger^®^, 100% fruit content, manufactured for ALDI Nord, Essen, Germany; 30 g/L Agar-Agar bacteriological from Carl Roth GmbH & Co. KG, Karlsruhe, Germany) or in a 5% orange juice medium [12] at 160 rpm and 20–22 °C. All inoculation cultures had an age of 7 days before transferring into the experimental set-up. The 5% orange juice medium (mixture of 100% orange juice und tap water) used was analyzed concerning the main ingredients. It contains the carbohydrates glucose (1.54 ± 0.2 g/L), sucrose (0.94 ± 0.1 g/L) and fructose (1.78 ± 0.1 g/L). The total concentration of reduced sugar amounts to 3.8 ± 0.04 g/L. The total nitrogen content was measured to 39.60 ± 0.1 mg_N_/L and the organic acids were analyzed to 320.5 ± 16.2 mg/L. The orange juice medium used has a natural pH value of 4.

### 2.1. Temperature Dependency of Growth and Xylindein Production of Chlorociboria aeruginascens

To test the influence of temperature on the growth and xylindein production of *C. aeruginascens*, five temperatures (6, 14, 20, 24, 28 °C) were applied to the fungal cultures in 4 replicates at each temperature. As a growth medium, orange juice agar was used in 92-mm petri dishes (Sarstedt, Nümbrecht; Germany). The orange juice agar contained 50% orange juice (Sonniger^®^, 100% fruit content, manufactured for ALDI Nord, Essen, Germany) and 30 g/L agar-agar (Agar-Agar bacteriological; Carl Roth GmbH & Co. KG, Karlsruhe, Germany). The stock solutions were autoclaved separately at 121 °C for 20 min for sterilization.

The agar plates were inoculated with blue–green fungal culture, which was pre-cultivated on 50% orange juice medium. The inoculum was placed in the middle of the agar plates and incubated at the above-mentioned temperatures in the dark. The measurement occurred weekly for a duration of 8 weeks. The radius of growth beginning with the edge of the inoculum was measured. The radius was measured in 4 directions (above, left, bottom, and right) for each replicate (*n* = 4 plates* 4 directions = 16).

### 2.2. Influence of pH Values on Xylindein Stability, Biomass and Xylindein Production

#### 2.2.1. pH Stability of Xylindein Extracts

To evaluate the pH stability of the extracted xylindein, two different solvents were used for the preparation of the pigment extracts. Filtrated (glass microfiber filters, 0.7 µm, VWR International) and water-washed discolored biomass from a 14-day-old liquid shaking culture from 5% orange juice medium was used as the extraction material. The dried biomass was crushed using an ultracentrifugal mill (ZM 200) equipped with a 12-tooth rotor (Part No. 02.608.0041) and a ring sieve with a 0.5-mm aperture (Part No. 03.647.0257, all: Retsch, Haan, Germany). A total of 500 mg of colored biomass prouder was mixed with 5 mL of the solvents, dichloromethane (DCM) [5,14] or acetone [14], at room temperature for 5 h using an overhead shaker. To separate the solids, the mix was centrifuged (7200 *g* for 5 min) afterwards.

Three different buffer systems were chosen for the preparation of the examined pH values; for pH 1 to 6, a citrate buffer; for pH 7 and 8, a citrate phosphate buffer; for pH 9 to 13, a glycol buffer. A volume of 100 µL of the pigment extract was mixed with 1 mL of the appropriate buffer to adjust the pH values from pH 1 to 13.

The absorbance spectrum (from 250 to 800 nm) of the discolored growth media, the xylindein containing the DCM extract as well as the pH-manipulated acetone extracts was measured by using a spectrometer (DU 640, Beckman Coulter, Indianapolis, USA). The presence of xylindein in the filtrated culture medium was determined by the detection of the two xylindein typical peaks between 600 and 700 nm, as reported in [3,5,15].

#### 2.2.2. Influence of pH on Biomass and Xylindein Production During Liquid Shaking Cultivation

To investigate the influence of the pH on biomass and xylindein production, a cultivation was prepared in Erlenmeyer flasks. Therefore, 16 100-mL Erlenmeyer flasks were filled with 50 mL of 5% orange juice medium. The pH values were adjusted in the range of 2 to 9 using NaOH and HCl. The flasks were shaken at 150 rpm and cultivated at 22 °C. To quantify xylindein production, the xylindein diffused into the liquid culture was measured. Therefore, the absorbance spectrum in the range of 250 to 800 nm was determined with a spectrometer (DU 640, Beckman Coulter, Indianapolis, USA). Therefore, the medium was centrifuged (7200× *g* for 5 min) to clean the liquid culture broth from biomass. When the liquid medium contains xylindein, two typical absorption peaks at 643 and 700 nm [3,12] occurred.

### 2.3. Xylindein Production by Chlorociboria aeruginascens Depending on Disolved Oxygen in the Liquid Growth Medium

A volume of 100 mL of 5% orange juice medium (Sonniger^®^, 100% fruit content, manufactured for ALDI Nord, Essen, Germany) was filled in each of the two 500-mL Schott glass bottles (Duran, Wertheim/Main, Germany). One bottle was closed with a membrane screw cap for oxygen transfer (O_2_+), the second bottle was closed with a usual screw cap for no air transfer (O_2_−). The medium in the bottles was autoclaved at 121 °C for 20 min. Afterwards, two inoculation plugs of an orange juice agar culture of *C. aeruginascens* with a size of 5 × 5 mm were transferred into each sterilized bottle. The bottles were prepared with a sensor spot of PreSens (Precision Sensing GmbH, Regensburg, Germany) to measure the oxygen partial pressure in the bottles. The culture was shaken at 120 rpm and 22 °C. The cultivation was performed for 18 days in two replications. The experiment was replicated twice with good reproduction.

### 2.4. Light Intensity Dependence of the Growth and Xylindein Production of Chlorociboria aeruginascens

For the light dependency investigation, the parallel cultivations system called Respiration Activity MOnitoring System (RAMOS^®^, from HiTec Zang GmbH; Herzogenrath; Germany) was used. The experimental set-up is specified in Anderlei [16] and Socher [17]. In the RAMOS^®^, high-power white light LEDs (CRI 90 + PowerBar LED, Nichia, Tokushima, Japan) are integrated. Different mean light intensities of the LED were measured exemplary by using a PAR light sensor (deka Sensor und Technologie GmbH, Teltow, Germany). During cultivation, the LEDs were adjusted to high light intensity (approximately 1300 µmol/(m^2^s)), low light intensity (approximately 1100 µmol/(m^2^s)) and no light.

In the cultivation system, 12 shaking flasks can be cultivated in parallel. Each flask contains 100 mL (work volume in 500-mL flasks) of 5% orange juice medium (Sonniger^®^, 100% fruit content, manufactured for ALDI Nord, Essen, Germany), which was autoclaved at 121 °C for 20 min for sterilization. Each flask was inoculated with two 5 × 5 mm inoculation plugs of an orange juice agar culture of *C. aeruginascens*. In the beginning, the initial oxygen content amounted to 20.95%. The flasks were shaken at 120 rpm at 22 °C.

Each light intensity was replicated 4 times, containing 2 measuring flasks, where the oxygen transfer rate (OTR), carbon dioxide transfer rate (CTR) as well as respiratory quotient (RQ) were measured. Two reference flasks were used to take samples to measure the absorbance spectrum (DU 640, Beckman Coulter, Indianapolis, USA) of the liquid culture medium. The experimental set-up was replicated twice with good reproduction.

## 3. Results and Discussion

### 3.1. Temperature Dependence of Growth and Xylindein Production of Chlorociboria aeruginascens

**Figure 1:**
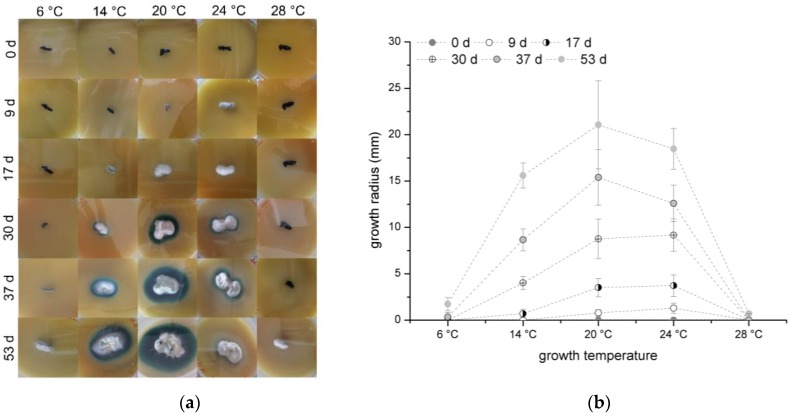
(**a**) Appearance of *Chlorociboria aeruginascens* on orange juice agar at different temperatures. (**b**) Growth radius over the growth temperature of *Chlorociboria aeruginascens* on orange juice agar at different temperatures (*n* = 16).

Each organism has a tolerance range for optimal growth parameters and, consequently, also for temperature. In general, wood populating fungi are mesophilic organisms and prefer temperatures in the range of 20 to 40 °C [13]. In the work presented here, a temperature range from 6 to 28 °C was tested over 53 days. For the test, *C. aeruginascens* was cultivated on orange juice agar and the growth radius was measured once a week. The results are presented in Figure 1a colony appearance and Figure 1b growth radius over the time.

In general, the fungus was able to grow at each tested temperature, but the growth rate differed. While fungal growth started with the formation of white mycelium with a light blue–green haze as surface hyphae, which transforms to a blue–green surface mycelium over time, the substrate hyphae of the whole colony created the typical blue–green color, which is caused by xylindein production.

In the experiments, 20 °C was determined as the preferable growth temperature for *C. aeruginascens* as shown in Figure 1b. Also, Richter and Glaeser determined that 22–24 °C is an optimal growth temperature [18]. In addition, in 1928, Frenzel [19] prepared similar experiments with the fungus *Chlorociboria aeruginosum*. He observed not only a dependency of growth rate but also on the color appearances of the mycelia at different temperatures in the range of 7 to 38 °C on different growth media. He found that the optimal growth temperature was 19 °C.

While the optimal growth temperature can be confirmed, the various color appearances depending on the temperature were not observed on orange juice agar. The different behavior of the cultures in Frenzel’s experiments and the ones presented here can be due to using different fungal strains and therewith caused by biological diversity. Also, the usage of different growth media, which have a high influence on xylindein production [10], can be the cause of differences.

Furthermore, Frenzel [18] reported that the fungus had the same growth radius at 14 °C as it did at 25 °C. In our own experiments, the growth radius at 14 °C is smaller than at 24 °C for the cultivation on orange juice agar over time as shown in Figure 1b. Of particular note is the different sizes of the pigmented zone. The intensified pigmentation at 14 °C could be the reason for a higher spore concentration in the orange juice agar. *C. aeruginascens* belongs to the class of ascomycetes and, consequently, they are able to produce spores as mycelium cultures. From studying other fungi, it is known that lower temperatures, for example 14 °C, induce the formation of fruit bodies because it simulates the colder temperatures in fall, where fungi strive for reproduction [13]. As a result, the higher pigmentation of *C. aeruginascens* might be a reaction to the lower temperature by releasing blue–green spores.

*C. aeruginascens* started to grow after 37 days at 6 °C and 53 days at 28 °C. The metabolism and the growth were inhibited by the either too low or rather too high temperatures. The late growth can be explained by the mechanism of survival.

In summary, the optimal growth temperature for *C. aeruginascens* was determined at 20 °C and the growth is limited at a temperature lower than 6 °C and higher than 28 °C.

### 3.2. Influence of pH Values on Xylindein Stability, Biomass and Xylindein Production

#### 3.2.1. pH Stability of Xylindein Extracts

The influence of various pH values on the target product xylindein was tested. Therefore, the two solvents, DCM and acetone, were used.

To detect and investigate pigments, different methods are available. Xylindein (C_32_H_24_O_10_, 568.534 g·mol^−1^ [7]) enables, due to its blue–green color, the use of color analysis methods for detection and quantification, such as LAB or UV–Vis spectrometry [3,5,14,15]. The LAB method is often used, but mostly only the deltaE values were presented in literature. The deltaE value gives some information about the color shift without color direction and not about the color itself. For comparison, a and b values would be more interesting.

In our laboratories, we used the absorbance spectrum to detect xylindein (spectrometry). Because it enables a measurement of a production parameter such as intensity or yield in liquid media (filtrated culture medium at the end of cultivation). In the preparatory work, we were able to show that an increase in the absorbance at a wavelength range of 660 nm is a sign of increasing xylindein production. However, our calibration could only be performed with extracted xylindein, as a commercial standard is not available.

**Figure 2:**
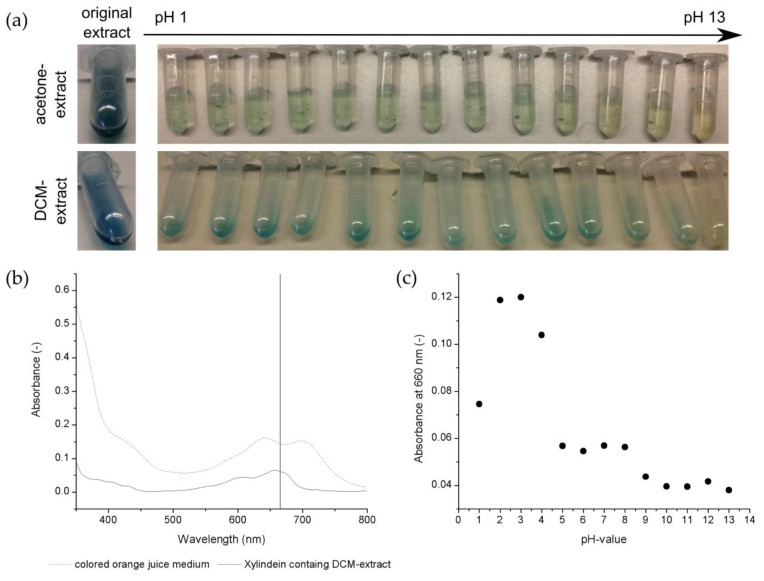
(**a**) Xylindein containing acetone as well as DCM extracts over various pH values (pH 1 to 13). (**b**) Absorbance spectrum from 350 to 800 nm from colored cultivation medium of a *Chlorociboria aeruginascens* culture in 5% orange juice. (**c**) Absorbance at 660 nm of the tested pH-manipulated xylindein containing acetone extracts.

Firstly, the absorbance spectrum of the *Chlorociboria aeruginascens*-discolored growth medium (orange juice) and the xylindein containing DCM extract are compared in Figure 2b. In the absorbance spectrum for the discolored growth medium as well as that for the DCM extract, two xylindein typical peaks are detectable [3,12]. The peaks, represented by the absorbed wavelength, are shifted regarding the culture medium and the DCM extract. Also, a shift from a blue–green (discolored orange juice medium) to blue (xylindein containing the DCM extract) color was observed. These phenomena result in the properties of the used solvent. When the solvent evaporates, the resulting product changes back to a blue–green color. The wavelength of 660 nm was chosen as a reference wavelength for all further experiments to measure the presence of xylindein in the growth medium. In Figure 2b, this wavelength represents the xylindein typical wavelength in the medium, as well as in the extract.

Figure 2a shows pictures of the pH-manipulated acetone extracts as well as the DCM extracts. The DCM extracts did not mix with the added buffer because two separate phases were visible as a result of the different polarities of the liquids. The effect of the buffer and, thus, the pH on the extract was only possible at the phase interface. Therefore, the absorbance of these samples was not measurable. Also, no color change in the samples over the pH shift from 1 to 12 was visible. However, the instability of the xylindein containing DCM extract was detected at pH 13 with the naked eye, because of the bleaching of the sample.

For the xylindein containing acetone extracts, the absorbance was measurable. The xylindein typical peaks in the wavelength range from 600 to 700 nm were determined for the samples at pH 1 to 8. The intensity of the measured absorbance varied in this pH range, which is demonstrated in Figure 2c. Here, the absorbance at 660 nm over the adjusted pH values in the xylindein containing acetone extracts is illustrated. The highest absorbance at 660 nm was measured at pH 2 and 3. At lower as well as higher pH values until pH 8, the absorbance decreased. Hence, the pigment-containing extract probably slowly decayed. For pH-manipulated samples with a pH higher than pH 9, non-xylindein typical peaks were detected. Hence, the xylindein is not stable for those pH values or rather the color change in the acetone as well as DCM extracts is a result of the accumulation of water around the OH groups of the xylindein. This is measurable with the absorbance spectrum and a shift of the absorbing wavelength (shift approx. 30 nm). The color appearance changes slightly between the original acetone extract and the pH-manipulated sample, which is a result of the dilution (only the intensity (absorbance) differs, no wavelength shift is detectable at same color).

#### 3.2.2. Influence of pH on Biomass and Xylindein Production During Liquid Shaking Cultivation

The pH value not only influenced the stability of the target product xylindein, but each organism also has a tolerance range of pH values and a moisture content range for optimal growth. In general, soft rot fungi prefer high moisture contents [20] and low pH values in the range of 3.3 to 6.4, but they are also able to grow in an environment with pH values up to 11 [13]. Furthermore, fungi are able to change the pH value of substrates by consuming nutrients and by producing primary and secondary metabolites [13].

In preliminary experiments, the preference of *C. aeruginascens* in the acidic range has already been shown. Now, the preferred pH value for growth and xylindein production should be determined in detail. For that, *C. aeruginascens* was cultivated in liquid medium (double determination) at a pH range of pH 2 to 9. Xylindein production was measured by the absorbance spectrum from the liquid culture medium. This is possible through the fact that xylindein diffuses into the medium with increasing xylindein production and culture time, as already described by Weber et al. [3]. The complete xylindein content is not detectable using this method, but the signals can be compared in relation to each other. To quantify xylindein directly, it must be extracted from the fungal biomass and the medium. This requires larger amounts of biomass for a reproducible determination and can only be achieved in larger cultivation scales.

**Figure 3:**
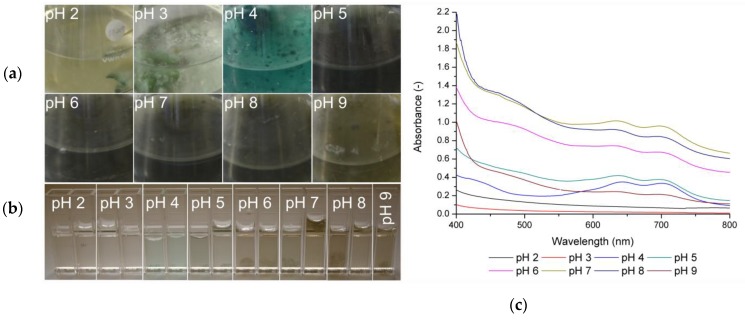
(**a**) Appearance of *Chlorociboria aeruginascens* in culture flasks cultivated in 5% orange juice at different pH values in the range of 2 to 9. (**b**) Cuvettes filled with liquid culture medium of the different flasks from (**a**). (**c**) Absorption spectrum of the liquid culture medium of *Chlorociboria aeruginascens* in orange juice at different starting pH values (*n* = 2).

The results of the various cultivations are shown in Figure 3. In the left upper section of the pictures (a), culture flasks with 5% orange juice (composition, see Section 2. Material and Methods) at different pH values during cultivation are given. The lower part of Figure 3b presents cuvettes with the liquid culture medium in lower thicknesses. In the pH range between 5 and 9, the biomass, as well as liquid media, changed its color to dark brown. The culture at pH 3 and 4 showed biomass growth as well as blue–green pigmentation of biomass, with a higher intensity of the color at a pH of 4. The pH 2-culture did not show significant biomass growth or pigmentation.

In Figure 3c, the absorbance spectrum of the liquid culture medium is given as an indication of the diffusion of pigments. If xylindein is in the liquid media, the absorption spectrum shows two characteristic peaks between 643 and 700 nm [3,12]. The measured color appearance in this wavelength range rises across the pH values from 4 to 8 (Figure 3c) but, at the same time, the absorption at other wavelengths increases too, which is an indication of the production of different other metabolites besides xylindein. The color of the culture medium was brown to almost black, which is detectable by the absorption spectrum where all visible wavelengths were absorbed by the produced metabolites.

No xylindein typical peaks were detected in the culture at pH 2, which is a result of no biomass growth, and pH 3. For pH 3, good biomass growth and biomass pigmentation was visible, but the saturation was lower than for pH 4. It is possible that the fungus did not release any pigmented material into the culture medium at the time of cultivation. Hence, xylindein is not detectable in the growth medium. Surprisingly, a xylindein production was measurable in the pH 9 cultivation. The absorbance was lower than the pH range of 4 to 8, but typical xylindein peaks were detectable. In Figure 2a, xylindein containing acetone extract did not show stability at pH 9, but xylindein was stable during cultivation. It can be presumed, that the fungus lowers the pH value of the culture medium to survive. The lower absorbance might be a result of a lower stability of the pigment itself.

Frenzel [18] also prepared pH-dependence experiments in agar-based systems. There, he did not detect any growth over a pH value of 8 and pigment production over a pH value of 6.7. In the work presented, a low metabolic reaction, as well as pigment production, were observed by means of color change at higher pH values during liquid cultivation. Tudor et al. [21] also prepared pH experiments focusing on pigmentation. They found that various fungi reacted by pigmentation at certain pH values in agar-based cultivation systems. For *C. aeruginascens*, they observed a similar behavior at a pH range of 2 to 7. For higher pH values, the fungus did not show any pigmentation just as Frenzel [18] described. In this study presented, a liquid culture was used and the pigmentation of the fungus at a pH over 8 was observed. The difference between the experiments by Frenzel [18] as well as Tudor et al. [21] and our investigations is the cultivation system (solid medium vs. liquid medium) as well as the fungi available nutrients used.

In summary, *C. aeruginascens* produces xylindein with fewer other visible colored metabolites at a pH of 4 as given by Figure 3c. Biomass growth is limited at a pH lower than 2 and higher than 9. 

### 3.3. Xylindein Production by Chlorociboria aeruginascens Depending on Disolved Oxygen in the Liquid Growth Medium

As many wood-colonizing fungi, *Chlorociboria* sp. is an aerobic organism. Thus, oxygen availability is essential for survival and growth. As already described in [10,22], limitations can also switch on certain metabolic pathways. Hence, in this experiment, whether oxygen limitation as a stress factor has a positive effect on xylindein production was investigated. The cultivation of *C. aeruginascens* in our experiments was performed both with and without air transfer. Frenzel [18] described an experiment with *Chlorociboria aeruginosum,* where he tried to cultivate the fungus without oxygen in different agar media but because of his preparation technique, the fungus was not in a completely air-tight environment for all replicates.

He found that the colonies of fungi with more oxygen had a bigger size in diameter than colonies with less available oxygen. Frenzel did not give information about the influence of oxygen on pigment production. In his results, Frenzel described that the fungus needs oxygen and if the oxygen concentration lowers, the growth reduces. Furthermore, if the fungus is under exclusion of air for short periods, there is no damage to the fungal culture [18].

**Figure 4:**
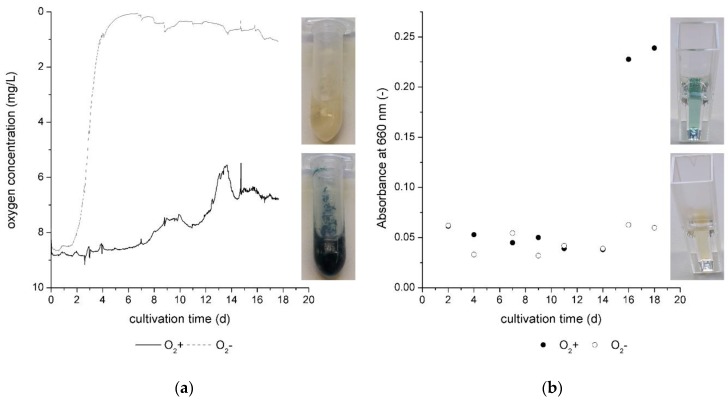
(**a**) Online measurement of oxygen concentration during the cultivation of *Chlorociboria aeruginascens*. (**b**) Absorbance at 660 nm of the liquid culture medium during cultivation with and without oxygen. (*n* = 2).

In Figure 4a, the oxygen concentration is given over the cultivation time for cultivation without oxygen limitation (O_2_+ ) and with oxygen limitation (O_2_−). The oxygen level decreased approximately to 0 mg/L in the first 6 cultivation days in the cultivation without continuous air transfer. Hence, the fungus consumed the total dissolved oxygen from the nutrient broth.

For O_2_+ cultivations, the oxygen concentration decreased slowly in the beginning of the cultivation. Oxygen consumption is a sign of the beginning of biomass growth and, as of day 6, the oxygen concentration decreased with a higher slope. At this point of the cultivation, the exponential growth phase started. At the same time, in the O_2_- cultivation, the oxygen concentration slightly increased. This was noticed in the sampling on days 2, 4, 7 9, 11, 14, 16, and 18 of the cultivation. While the cultivation system was closed and the oxygen level was controlled during cultivation, it was shortly opened for sampling. The cultivation jars for O_2_− cultivation were not flushed with nitrogen or another inert gas. Hence, it was possible that the oxygen level increased slightly in the jars during sampling. The effect is also given by Figure 4a, indicated by small peaks on the day of sampling.

The curve shape of the oxygen concentration during the O_2_+ cultivation allows us to assume that the fungal growth is separated into two different nutrient sources as observed and also mentioned in 3.4. for the light dependency cultivation. On day 11 of the O_2_+ cultivation, the biomass started to change from a white to blue–green color. On the same day, the oxygen consumption in the culture medium started to rise again with a higher slope. Hence, the metabolism changed for the benefit of xylindein production. For this cultivation phase (from 11), the oxygen consumption was higher than for the first cultivation phase (day 6 to day 11). As illustrated in [10] acetyl-CoA is the initial molecule for the polyketide pathway, which can be used by *C. aeruginascens* for the production of xylindein [4]. Aerobic organisms utilize oxygen for the production of acetyl-CoA by the oxidative decarboxylation of pyruvate from glycolysis and keto acids from protein catabolism as well as the β-oxidation of the lipid metabolism.

Xylindein is verifiable in the liquid growth medium on day 16 of the cultivation as given by Figure 4b. At this point, the oxygen concentration did not change significantly in the culture medium, which was seen in the stationary growth phase of the fungus. The measured pigment in the liquid culture medium also did not increase significantly, which supports the statement about the stationary growth phase of the fungus.

In summary, oxygen limitation has no positive effect on pigment production in biomass or liquid media (Figure 4b) by *C. aeruginascens*. Instead, a sufficient supply of oxygen is necessary.

### 3.4. Light Intensity Dependency of the Growth of Chlorociboria aeruginascens

Fungal mycelia practice cell respiration. The oxygen transfer rate (OTR) and carbon dioxide transfer rate (CTR), as well as the respiratory quotient (RQ), are useful parameters to determine the growth habits of different organism during cultivation [16,17].

The light dependency investigation was performed in a Respiration Activity MOnitoring System (RAMOS^®^). The growth habits of the fungus represented by the OTR, CTR, and RQ were determined as online measurements during cultivation. The cultivation system gives the opportunity to cultivate one reference flask for each measuring flask. The reference flask was used to determine xylindein production in the growth media, while the measuring flask was only used for the online measurement of the OTR, CTR, and RQ.

**Figure 5:**
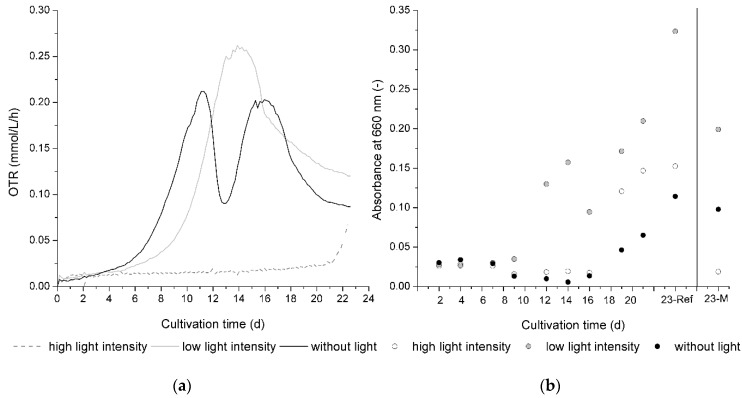
(**a**) Online measurement of the oxygen transfer rate for a light-dependent cultivation (without light, low light intensity, high light intensity) of *Chlorociboria aeruginascens*. (**b**) Absorbance at 660 nm of the liquid culture medium. Ref—reference flasks; M—measuring flask (*n* = 2).

As given by Figure 5, the growth of *C. aeruginascens* and also xylindein production is significantly influenced by light. Figure 5a presents the OTR progression during cultivation for all three different tested light conditions. Figure 5b shows the mean absorption at 660 nm of the liquid culture medium of the reference flasks over the cultivation time.

The experiment was prepared twice with the same set-up (two replicates per run), only the age of the pre-culture was different. The curve shapes, as well as the pigmentation results, were reproduced in both replicates and runs. The exponential growth started slightly earlier than in the second cultivation with the mycelium which was 3 weeks older only during the first cultivation, where the younger mycelium was used. This is a common effect which is already known in other microorganisms [23]. However, the time lapse between the different beginnings of the exponential growth phase also depends on light intensity (difference between first and second cultivation without light accounts for 1 d; for low light intensity, accounts for 2 d; for high light intensity, accounts for 5 d). The results presented in Figure 5a,b are descended from the first run.

A small difference between the flasks with the same light conditions was also determined, which is also presented in Figure 5b, where the absorbance of the liquid culture media on day 23 varied between the measuring (23-M) and reference flask (23-Ref). This effect is attributed to the light source itself. For the experiment, LEDs were used. It is well known that LEDs have a certain lifetime. Hence, the light intensity, which was measured in the beginning of the experiment, is only a rough estimation for high light intensities and low light intensities, because different LED spots were used for each flask. To minimize the LED lifetime influence on both replicates of cultivation, the same flask position or LED spot was used for the same light intensity in both the first and second cultivation. As such, the quality of the data was secured.

In bioprocess engineering, the OTR is a parameter that is often used. It describes the oxygen consumption of organisms and therewith verifiable statements can be derived for biomass growth during a cultivation process. As shown in Figure 5a, the beginning of biomass growth given by the exponential growth phase started in dark flasks earlier than in lighted flasks. Therefore, the light intensity has a significant influence. While the difference between the beginning of the exponential growth phase of the flasks with low light intensity and without light is smaller than the difference between the beginning of the exponential growth phase of the flasks with low light intensity and high light intensity, the exponential growth phase started for high light intensities when the experimental time ended. The cultivation was aborted because the assertion regarding the light intensity dependence of biomass growth and pigment production was shown at this time.

Furthermore, the curve shape of the dark cultivation represents a typical two-parted metabolism for *C. aeruginascens* as investigated by Stange et al. [10]. Here, the biomass increases in a first growth phase by using available sugars such as glucose and the nitrogen source in the orange juice. On day 11 of the cultivation, the OTR achieved its maximum, which means the biomass growth had its maximum slope. Between day 11 and 13 of the cultivation, the OTR decreased. This means that biomass growth slowed down. When the OTR increased, the metabolic activity started again. During this phase, the fungus rearranges its metabolism and passes over into the second part of the growth. On day 12 of the cultivation, the biomass started to change from a white to blue–green color. By Stange et al. [10], it was observed that the biomass changes its color under nitrogen limitation. When *C. aeruginascens* produces xylindein, it has to change its metabolism from a nitrogen-available growth to a nitrogen-limited growth. The nitrogen limitation and the rearrangement of the metabolism for the benefit of xylindein production were presented by this typical curve shape.

Figure 5b shows the absorbance at 660 nm of the liquid culture medium to evaluate the release of fungal pigments from the mycelia pellets. It was observed that the release of the pigments also depended on the applied light conditions. While the biomass of *C. aeruginascens* changed from a white to blue–green color on day 12 of the dark cultivation, the pigment xylindein was firstly determined in the liquid growth media from day 19 of cultivation as given by Figure 5b. Arguably, the balance of xylindein and biomass, which is primarily created by metabolizing the nitrogen and carbohydrate sources, is shifted to xylindein production. For instance, on day 19 of cultivation, the saturation of the biomass with pigment was reached and the fungus delivered the pigment into its environment.

The divided growth behavior, which *C. aeruginascens* shows during cultivation without light, is not obviously shown during cultivation with less light intensity. While the exponential growth phase started later here than for the cultivation without light, the pigmentation of biomass started at the same time (day 12) with a much higher saturation of xylindein. As illustrated in Figure 5b, the pigment can be documented in the liquid culture medium here on the same day (in biomass: day 12; in culture medium: day 12), which is much earlier than in dark cultivation (in biomass: day 12; in culture medium: day 19). That means that the metabolism of the first and second cultivation phases during light exposure does not take place one after the other but rather is superposed.

With a high light intensity (approx. 1300 µmol/(m^2^s)), biomass growth was very low, and the exponential growth phase started on day 20. At the same time, pigment production began. The biomass showed a high saturation, far higher than the biomass of dark cultivation. On the same day, xylindein can be determined with the spectrometer in the liquid culture medium.

The terms high intensity and low light intensity are relative in this case. The mean light intensity on the earth’s surface amounts to approx. 1560 µmol/(m^2^s) and the light intensity at the equator on a day without clouds amounts to approx. 4700 µmol/(m^2^s) [17].

Summarizing, it was possible to prove the relation between light intensity, growth, and xylindein production of *C. aeruginascens*. In general, it can be stated that the lower the light intensity, the earlier the exponential growth of the fungus starts. In addition, if the culture is exposed to light, the xylindein yield is higher.

Because fungi are heterotrophic organisms, their metabolism is usually not dependent on light. However, Schmidt [13] reports that fungal mycelium is able to respond to light by the production of spores. Light with short wavelengths (like blue light) supports this effect, while light with long wavelengths inhibits this effect. The LEDs used were covering a spectrum from 420 to 760 nm with a small amount of blue light [17]. *C. aeruginascens* belongs to the class of ascomycetes and therewith they are able to produce spores as mycelium culture. A blue–green-discolored liquid culture medium was examined by light microscopy. One part of the pigment xylindein is bound to the biomass of the filamentous fungus. It was observed that another part of the pigment was bound to blue–green spores, which were spread into the liquid media. The blue–green spores can also be filtrated by ultrafiltration, with a clear yellowish medium as filtrate.

Schmidt [13] also reports on the microbial pigment production of, for instance, *Aspergillus niger*, as protection against irradiation. The pigment xylindein is known in restoration for its great UV-stable properties [3,13,14,15,24]. Even intarsia from the 15^th^ century shows great colorfastness and lightfastness [1,15]. It might bring the relation of the UV stability of the pigment xylindein to light-dependent growth and pigment production behavior, which is presented in this work. It was observed that the biomass and pigment production, and there with the metabolism, of *C. aeruginascens* is affected by light and light intensity. One might argue that light is a stress factor for *C. aeruginascens* and, therefore, a trigger to change the metabolism from biomass growth to pigment production assisted by a lower growth metabolism conceivably as UV protection. Thus, light limits growth metabolism but promotes xylindein metabolism. *Chlorociboria* sp. is often found on wood located under fallen leaves. This fact confirms an optimal growth in dark or lower light intensities.

## 4. Conclusions

The fungus *Chlorociboria* sp. is well known as spalting fungi, which causes a blue–green discoloration in wood [1]. Spalting is an effect on various environmental influences (competition, nutrient supply, abiotic factors) and, thus, a stress repercussion. However, in the common literature, the specific stress factors for *Chlorociboria* sp. and xylindein production are not explicitly presented. Our experiments were performed to evaluate environmental factors such as stress factors and their influence on biomass growth as well as xylindein production.

The pigment xylindein appears to be a special survival molecule for *Chlorociboria* sp. as displayed in this work. The production of xylindein started earlier for cultivation with light where, arguably, xylindein is produced by the fungus as UV protection. Also, in cultures with pH values, which are not comfortable for biomass growth, *Chlorociboria aeruginascens* produced, besides various other secondary metabolites, xylindein. Alone, oxygen limitation had a negative impact on xylindein production. No pigmentation was visible, which results in non-metabolic activity of the fungus.

A delay was also detected at the start of biomass production when there are no optimal growth conditions. The lag phase depends on the growth condition and, therewith, the start of biomass and xylindein production. For non-optimal growth conditions, the lag phase lasted longer than for optimized growth conditions, which means that the fungus has a high resistance against adverse environmental conditions proven by lag phases of more than 20 days for the light dependency investigation. Xylindein plays an important role as a protection molecule for the survival of this slow-growing fungus.

The optimized growth conditions were determined as a temperature of 20 °C, a pH value of 4, dark and aerobic cultivation.

Summarizing the results presented here with the results of [12], a xylindein production decoupled from growth by *C. aeruginascens* was identified. The fungus reacted with the production of xylindein as a result of A) nitrogen limitation in the substrate [12] and B) for growth unfavorable environmental conditions (e.g., light intensity and pH value).
